# Belowground Interactions Impact the Soil Bacterial Community, Soil Fertility, and Crop Yield in Maize/Peanut Intercropping Systems

**DOI:** 10.3390/ijms19020622

**Published:** 2018-02-22

**Authors:** Qisong Li, Jun Chen, Linkun Wu, Xiaomian Luo, Na Li, Yasir Arafat, Sheng Lin, Wenxiong Lin

**Affiliations:** 1 College of crop Sciences, Fujian Agriculture and Forestry University, Fuzhou 350002, China; liqisong0591@gmail.com (Q.L.); luoxiaomian01@163.com (X.L.); 2 Fujian Provincial Key Laboratory of Agroecological Processing and Safety Monitoring, College of Life Sciences, Fujian Agriculture and Forestry University, Fuzhou 350002, China; chenjunfafu@163.com (J.C.); wulinkun619@fafu.edu.cn (L.W.); lina20101020@163.com (N.L.); stanadar2012@gmail.com (Y.A.); 3 Key Laboratory of Crop Ecology and Molecular Physiology (Fujian Agriculture and Forestry University), Fujian Province University, Fuzhou 35002, China

**Keywords:** soil nutrition, soil bacterial community, microbial diversity, intercropping, T-RFLP, qPCR

## Abstract

Intercropping has been widely used to control disease and improve yield in agriculture. In this study, maize and peanut were used for non-separation intercropping (NS), semi-separation intercropping (SS) using a nylon net, and complete separation intercropping (CS) using a plastic sheet. In field experiments, two-year land equivalent ratios (LERs) showed yield advantages due to belowground interactions when using NS and SS patterns as compared to monoculture. In contrast, intercropping without belowground interactions (CS) showed a yield disadvantage. Meanwhile, in pot experiments, belowground interactions (found in NS and SS) improved levels of soil-available nutrients (nitrogen (N) and phosphorus (P)) and enzymes (urease and acid phosphomonoesterase) as compared to intercropping without belowground interactions (CS). Soil bacterial community assay showed that soil bacterial communities in the NS and SS crops clustered together and were considerably different from the CS crops. The diversity of bacterial communities was significantly improved in soils with NS and SS. The abundance of beneficial bacteria, which have the functions of P-solubilization, pathogen suppression, and N-cycling, was improved in maize and peanut soils due to belowground interactions through intercropping. Among these bacteria, numbers of *Bacillus*, *Brevibacillus* *brevis*, and *Paenibacillus* were mainly increased in the maize rhizosphere. *Burkholderia*, *Pseudomonas*, and *Rhizobium* were mainly increased in the peanut rhizosphere. In conclusion, using maize and peanut intercropping, belowground interactions increased the numbers of beneficial bacteria in the soil and improved the diversity of the bacterial community, which was conducive to improving soil nutrient (N and P) supply capacity and soil microecosystem stability.

## 1. Introduction

Intercropping is becoming common in the Americas, Asia, Africa, and Europe. It plays an important role in maintaining farmland ecosystem biodiversity and stability, improving resource efficiency, and achieving high and stable yields in the agroecosystem [1,2]. Most intercropping systems (i.e., maize/peanut, wheat/maize, faba bean/wheat, etc.) show yield advantages. However, some intercropping systems show yield disadvantages due to strong interspecific competition [3]. It is very necessary to explore the underlying mechanisms of yield advantages under intercropping systems. 

Intercropping involves the aboveground and belowground interaction of crops. In previous studies, the aboveground interaction in intercropping systems altered the canopy micro-ecology, resulting in improved solar light use efficiency and soil water storage capacity, and a reduction in the evaporation of soil moisture [4,5]. In recent years, more studies have been focused on soil nutrients and root exudates [6]. In legume/cereal intercropping systems in alkaline calcareous soils with low phosphorus, legume root exudates including malic acid and citric acid have been shown to acidify the rhizosphere and mobilize insoluble P while improving legume nodulation and nitrogen fixation [7]. Meanwhile, increased rhizosphere phosphorus availability was also observed in durum wheat and chickpea intercropping in neutral soil, but rhizosphere acidification was not observed; in fact, the pH value increased [8]. Moreover, in acidic soil, He et al. [9] suggested that the increase in plant P uptake was due to the changes in the microbial community composition in maize/chickpea and maize/soybean intercropping systems. Therefore, belowground interspecific interactions are complex, and further research is needed.

Soil microorganisms play crucial roles in the rhizosphere ecosystem, being involved in soil nutrient cycling, suppression of soil-borne pathogenic microorganisms, and the decomposition of organic matter, which is closely associated with the aboveground performance of plants [10,11]. In recent years, the root-associated microbial community has been the focus of many plant studies. Mortel et al. [12] found that *Pseudomonas fluorescens* was able to induce the salicylic acid signaling pathway in *Arabidopsis*, and further improved the resistance of plants to pests and diseases. Sanguin et al. [13] found that take-all decline disease in wheat was closely associated with the shift of the bacterial community in long-term monoculture. Weidner et al. [14] suggested that high soil microbial diversity was favorable for positive plant-soil feedback and nitrogen nutrient supply in soil.

Previous studies have demonstrated that plants can modify their rhizosphere microbial community through their root exudates [15,16], and that the shift of rhizosphere microbial can further affect the soil enzymes and soil fertility [17]. Therefore, the rhizosphere microbial community can be affected by different plants in intercropping, and the changes in soil microbial communities may play important roles in the benefits of intercropping. Intercropping of maize and peanut has been previously demonstrated to provide significant advantages, and has been commonly used [18,19]. However, little is known about the alternation of the rhizosphere microbial community at the species and genera level in the intercropping systems, and even less information is available about this in maize/peanut intercropping specifically. 

Molecular methods can assay the microbial community structure in phylum, genera, and even species in the rhizosphere soil of plants [20]. The terminal restriction fragment length polymorphism (T-RFLP) coupled with cloning sequences quantitative PCR (qPCR) technique offers the best possibilities for analyzing the structure and diversity of soil microbial communities [21,22]. In this study, maize and peanut were intercropped in three patterns of belowground interactions with the same aboveground canopy structure. Firstly, the changes in agronomic traits, photosynthetic characteristics, soil-available nutrients, and soil enzymes between different treatments were analyzed to explore the key factor (aboveground or belowground interactions) contributing to the yield advantage. Then, the shifts of soil bacterial community structure and functional diversity were assessed using qPCR and T-RFLP. The aim of this study is to explore the relationships between the changes of soil bacterial community and their related soil enzyme activities and yield advantage in maize/peanut intercropping regimes, in order to provide a theoretical basis and practical guide for reasonable intercropping and maintaining biodiversity in agricultural ecosystems.

## 2. Results

### 2.1. Yields in Field Experiments and Plant Properties in Pot Experiments 

The plant yields of two crops in field were measured in 2011 and 2012 under different treatments (Table 1). Two-year yield results showed that the maize yields using non-separation intercropping (NS) and semi-separation intercropping (SS) were significantly improved when compared with complete separation intercropping (CS) and monoculture treatment (MS). In 2011, the peanut yield was significantly higher when using NS and slightly higher when using SS and MS as compared to CS. In 2012, a similar trend was found in peanut yield between the treatments, and no significant differences were observed. Compared with monoculture, land equivalent ratio (LER) results showed yield advantages due to belowground interactions in the NS and SS patterns in the two-year experiments, while the intercropping pattern without belowground interactions (CS) showed a yield disadvantage.

In the pot experiment, the NS and SS patterns also showed the same trend when compared with CS (Table 2). The shoot biomass, root biomass, and net photosynthetic rate in maize with NS and SS were significantly higher than in maize with CS. Peanut root biomass, nodule number per plant, and dry weight per nodule were significantly higher in maize with NS and SS.

### 2.2. Soil Nutrition and Soil Enzyme Activities

**Figure 1:**
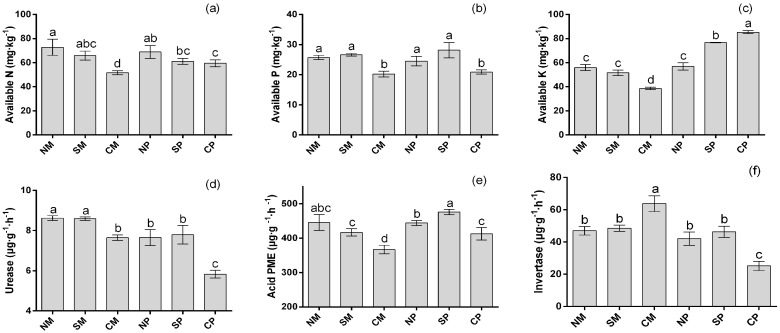
Soil available N (**a**); available P (**b**); available K (**c**); urease activity (**d**); acid PME (phosphomonoesterase) activity (**e**); and invertase activity (**f**) under different intercropping treatments. NM: non-separated maize; SM: semi-separated maize; CM: completely separated maize; NP: non-separated peanut; SP: semi-separated peanut; CP: completely separated peanut. Bars with different letters indicate significant differences (LSD test, *p* < 0.05, *n* = 3).

Differences in soil nutrient contents and soil enzyme activities were detected between the distinct treatments in the pot experiment (Figure 1). In maize rhizosphere soils, belowground interactions (in NS and SS) significantly enhanced soil-available nutrients (N, P and K), urease, and acid phosphomonoesterase (PME) as compared to CS. In peanut rhizosphere soils, the activities of soil urease, acid PME and invertase, and available P were significantly enhanced in the NS and SS treatments as compared with CS. Available N levels were significantly higher in the NS soil and slightly higher in the SS soil as compared to the CM soil. However, NS and SS reduced the available K contents in peanut soil as compared to the CS soil.

### 2.3. Shifts of the Soil Microbial Community

**Figure 2:**
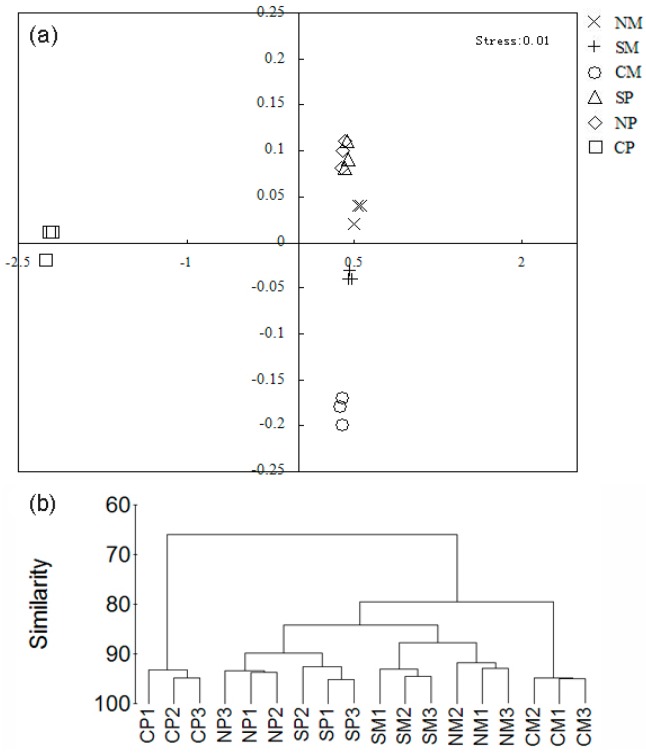
NMDS ordinations (**a**) and clustering analysis (**b**) of bacterial communities in soil. NM: non-separated maize; SM: semi-separated maize; CM: completely separated maize; NP: non-separated peanut; SP: semi-separated peanut; CP: completely separated peanut.

T-RFLP was used to analyze the rhizosphere bacterial communities of maize and peanut under different intercropping treatments. We obtained bacterial T-RFLP profiles using the digestion of four restriction enzymes (*Msp*I, *Hae*III, *Afa*I, and *Alu*I) (Figure S1). The terminal restriction fragments (T-RFs) of four enzymes were combined for matrix calculation. We analyzed the diversity of rhizosphere soil bacterial communities under different intercropping patterns (Table 3). Simpson and Shannon–Wiener indices were improved in plant soils under NS and SS as compared with CS. Meanwhile, the lowest value for CS was found in peanut. The result of non-metric multi-dimensional scaling (NMDS) and cluster analyses (Figure 2) showed that NS and SS crop soil bacterial communities in both plants clustered together and were considerably different from those of CS crops.

**Figure 3:**
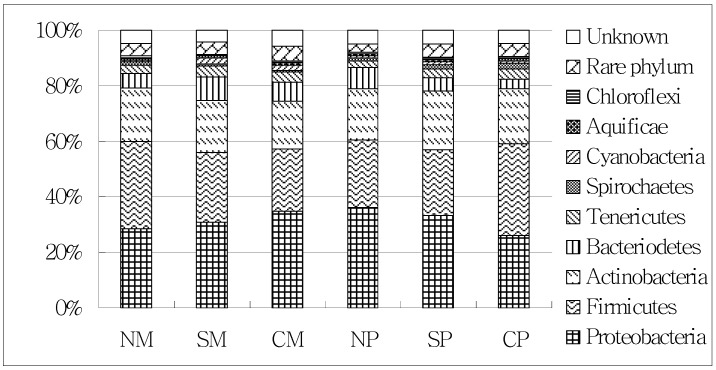
The groups of maize and peanut rhizosphere soil bacterial phyla under different intercropping patterns. NM: non-separated maize; SM: semi-separated maize; CM: completely separated maize; NP: non-separated peanut; SP: semi-separated peanut; CP: completely separated peanut.

Based on T-RFs, we grouped the identified bacteria into 11 phyla (Figure 3) using the phylogenetic assignment tool (PAT). Proteobacteria, Firmicutes, and Actinobacteria were the most abundant bacteria in the soil samples. Proteobacteria were the dominant bacteria in maize soil with CS. The presence of Proteobacteria gradually decreased in SS and NS maize soils, while quantities of Firmicutes and Actinobacteria gradually increased in maize soils with SS and NS. Firmicutes was the dominant bacteria in peanut soil with CS. The quantities of Firmicutes gradually decreased, while those of Proteobacteria gradually increased in peanut soils with SS and NS.

Furthermore, the similarity percentages (SIMPERs) of T-RFs was determined. NS and SS were combined as the planting pattern with belowground interactions (BI) and CS was the planting pattern without interactions (WI). Both maize and peanut were analyzed under these two patterns (BI and WI). The results showed that there was a dissimilarity of 19.33% between BI and WI for the maize soil bacterial community and 33.52% for peanut. The top T-RFs with 20% cumulative contribution to the dissimilarity are shown in Table 4 and Table S1. The results showed that under the BI pattern (SS and NS), numbers of beneficial bacteria (i.e., *Bacillus*, *Burkholderia*, *Pseudomonas*, etc.) were increased in maize and peanut soils. Among them, *Bacillus*, *Brevibacillus brevis*, and *Paenibacillus*, all of which belong to Firmicutes, mainly increased in the maize rhizosphere. *Burkholderia*, *Pseudomonas*, *Sphingomonas*, and *Rhizobium*, all of which belong to Proteobacteria, mainly increased in the peanut rhizosphere.

### 2.4. qPCR of Specific Bacterial Groups

**Figure 4:**
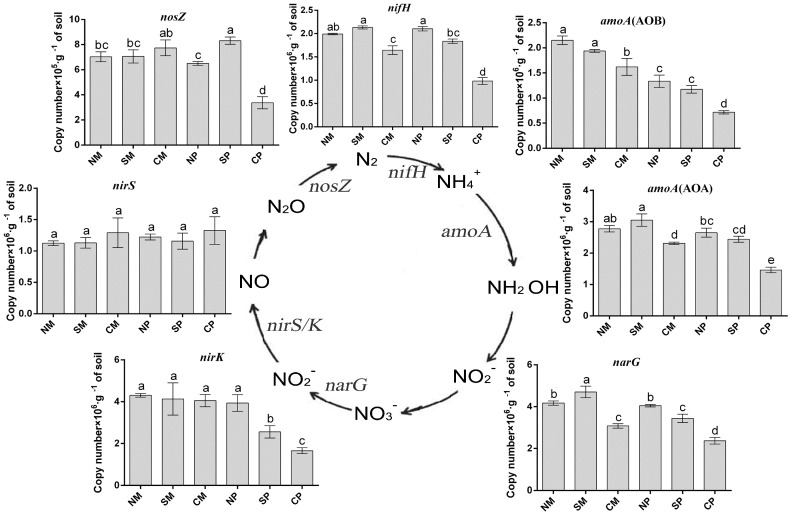
Quantification of genes involved in N-cycling from rhizosphere soil under different intercropping patterns. NM: non-separated maize; SM: semi-separated maize; CM: completely separated maize; NP: non-separated peanut; SP: semi-separated peanut; CP: completely separated peanut. Bars with different letters indicate significant differences (LSD’s test, *p* < 0.05, *n* = 4).

Effective use of nitrogen is considered the most important factor with respect to the advantages of intercropping. Based on previous results, soil-available N, urease activity, and the soil bacterial community was improved in the belowground interaction planting pattern. Therefore, seven genes encoding key enzymes in N cycling (*nifH*, *amoA* (AOA), *amoA* (AOB), *narG*, *nirK*, *nirS* and *nosZ*) were analyzed (Figure 4 and Figure S2). As for peanut, for *nirK*, there were no significant differences among the treatments, while abundances of the other six genes were significantly improved (*p* < 0.05) in conditions of belowground interaction through intercropping (NS and SS). In the maize rhizosphere, the results showed that the abundances of the nitrogen fixation-related gene (*nifH*), the ammonia oxidation-related gene (*amoA* (AOA), *amoA* (AOB)) and the nitrate reductase gene (*narG*) were significantly improved (*p* < 0.05) under the planting patterns with belowground interactions, while the other three genes (*nirK*, *nirS,* and *nosZ*) showed no significant differences.

**Figure 5:**
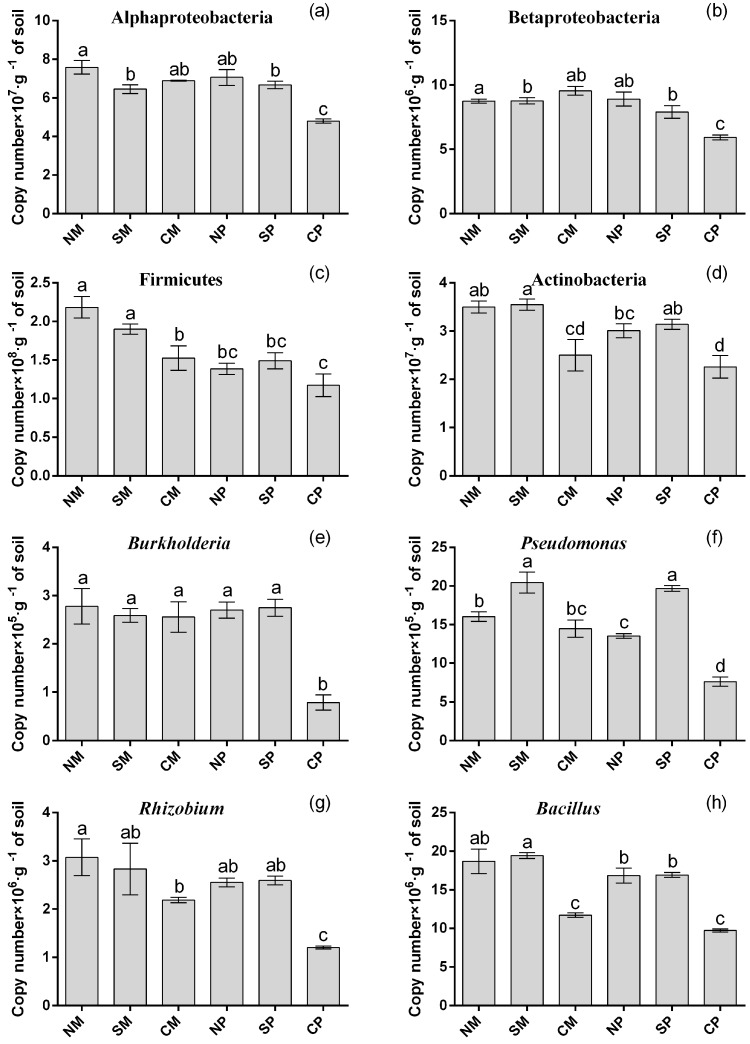
Quantification of genes involved in major microbial communities under different intercropping patterns. (**a**): Alphaproteobacteria; (**b**): Betaproteobacteria; (**c**): Firmicutes; (**d**): Actinobacteria; (**e**): *Burkholderia*; (**f**): *Pseudomonas*; (**g**): *Rhizobium*; (**h**): *Bacillus*. NM: non-separated maize; SM: semi-separated maize; CM: completely separated maize; NP: non-separated peanut; SP: semi-separated peanut; CP: completely separated peanut. Bars with different letters indicate significant differences (LSD’s test, *p* < 0.05, *n* = 4).

Quantitative PCR further confirmed the changes in the abundance of the main bacterial phyla (α-Proteobacteria, β-Proteobacteria, Firmicutes, and Actinobacteria) and beneficial bacteria (*Bacillus*, *Burkholderia*, *Pseudomonas*, and *Rhizobium*) in all six soil samples (Figure 5 and Figure S3). The numbers of *Bacillus*, *Burkholderia*, *Pseudomonas*, *Rhizobium*, α-Proteobacteria, and β-Proteobacteria were significantly higher in peanut soil under the planting pattern with belowground interactions. Meanwhile, significantly higher abundances of *Bacillus*, *Rhizobium*, Firmicutes, and Actinobacteria were observed in belowground interactions in maize soil. The results were consistent with the T-RFLP analysis (Figure 3 and Table 4).

## 3. Discussion

Legume and cereal intercropping has been widely applied in agriculture, and the mechanism of beneficial effects extensively studied [23]. According to previous studies, most intercropping systems display a yield advantage [6]. However, there is still no consensus on the underlying mechanisms of beneficial effects in intercropping regimes. In some works, the beneficial effects were attributed to the improvement of soil-available P by the acidification of root exudation (i.e., organic acids) in alkaline soil [7]. Meanwhile, other studies found that the improvement of soil-available P is not related to the pH value in acidic and neutral soils [8,9] because the organic acids secreted by the root are quickly fixed by the soil, and the concentrations of organic acids are too low (1~50) µM to activate insoluble P [24,25]. Hence, the advantages of maize/peanut intercropping need to be confirmed before studying their underlying mechanisms in a new soil environment. In a recent study, two-year LER in field experiments showed yield advantages in the presence of belowground interactions (NS and SS), while the without belowground interactions (CS) pattern showed yield disadvantages as compared to the monoculture. The results indicated that the advantages of maize/peanut intercropping were due to the belowground interactions, and the advantages may be derived from the enhancement of soil nutrient, enzymes activity and microbial community composition.

Soil enzymes are important bioactive proteins in soil that are mainly derived from microorganisms [17]. They directly participate in soil nutrient cycling and are closely related to soil fertility and soil environmental quality [26]. Urease participates in soil nitrogen cycling and indicates soil quality and fertility [27]. Phosphomonoesterase plays an important role in converting organic phosphate into inorganic phosphate, which can improve soil available P [28]. Invertase activitiy is correlated with soil carbon cycling, phosphorus content, microbial biomass, and soil respiration intensity [29]. Previous studies have demonstrated that interspecific interactions in intercropping systems could effectively improve the mobilization and uptake of nitrogen and phosphorus as compared to monocropping [8,30,31]. Higher soil enzyme activities can improve available N and P supply capacity in the plant soil. Our study found that urease, acid PME activities, and available N and P contents in both crops were significantly higher in the presence of belowground interactions (NS and SS) as compared to intercropping without belowground interactions (CS). Similar changes were observed in other intercropping systems [32,33,34]. Invertase were significantly enhanced in the NS and SS treatments as compared with CS in peanut soil, while decreased in maize soil. These results are supported by the finding of Dai et al. (2013), that the activity of invertase was significantly increased in peanut when intercropped with *Atractylodes lancea* [35]. The results indicated that intercropping of maize and peanut improved soil fertility through belowground interactions and the improvements were closely related to with the shifts in the soil microbial community, also indicating soil microbial community and enzymatic activity is sensitive to different plants’ interspecific interactions.

The microbial community is a key component of the rhizosphere soil ecosystem and can be used for evaluating soil quality [36]. Changes in soil microbial communities have been observed in mulberry/soybean, maize/chickpea, maize/soybean, and sorghum/peanut intercropping [9,37,38]. Our previous study demonstrated that Gram-positive bacteria (G^+^) were significantly improved in maize soils when intercropped with peanut, while Gram-negative (G^−^) bacteria were significantly improved in peanut soil [39]. Thus, the soil bacterial community in intercropping was studied by T-RFLP analysis and similar results were observed in the present study. The results showed that Proteobacteria (G^−^), Firmicutes (G^+^), and Actinobacteria (G^+^) were the most abundant in the different soil samples. Numbers of Firmicutes (G^+^) and Actinobacteria (G^+^) were increased in intercropped maize soils with belowground interactions (NS and SS), while Proteobacteria (G^−^) was increased in intercropped peanut. These trends were further confirmed by qPCR (Figure 5). Meanwhile, multi-dimensional scaling (NMDS) and cluster analyses clearly demonstrated that NS and SS soil bacterial communities in both plants clustered together and were considerably different from CS bacterial communities. 

Moreover, plant growth-promoting rhizobacteria have an important role in the agroecological system. They can suppress plant soil-borne pathogens, improve the concentration of available nutrients, and promote the growth of plants [40]. The SIMPER analysis of T-RFs delivered an insight into key variation bacterial between planting pattern with belowground interactions and without interactions. The result (Table 4 and Table S1) showed that higher abundances of beneficial bacteria (*Bacillus*, *Burkholderia*, *Pseudomonas* and *Rhizobium* etc.) were observed in belowground interaction intercropping treatments (NS and SS). *Bacillus*, *Brevibacillus brevis*, and *Paenibacillus* were found to have functions of phosphate solubilization, nitrogen fixation, suppression of pathogenic microorganisms, and improvement of legume nodulation. *Burkholderia* and *Pseudomonas* exist widely in healthy and disease-free soil, and they can effectively control the occurrence of soil diseases and improve the solubilization of fixed soil phosphorus (Table S1); *Sphingomonas* is involved in decomposition of aromatic compounds; *Rhizobium* is involved in nitrogen fixation and is closely related to peanut nodulation. We found numbers of *Bacillus*, *Brevibacillus brevis*, and *Paenibacillus*, belonging to Firmicutes (G^+^), mainly to be increased in the maize rhizosphere as a result of belowground interaction. Meanwhile, *Paenibacillus*, *Burkholderia*, *Pseudomonas*, *Sphingomonas* and *Rhizobium*, belonging to Proteobacteria (G^−^), were mainly increased in the peanut rhizosphere. In addition, *Bacillus*, *Burkholderia*, *Pseudomonas*, and *Rhizobium* were considered to be the key plant growth-promoting rhizobacteria [41] and were further analyzed by qPCR in six soil samples. The qPCR results were consistent with the T-RFLP analysis (Figure 5). The results revealed that belowground interaction in intercropping can induce changes in soil bacterial community structure and attract more beneficial bacteria, which participate in nutrient cycling, legume nodulation, and suppression of plant soil-borne pathogens. These findings explained the reason for higher soil nutrient (N and P) supply capacity and crop disease resistance in intercropping.

The level of soil bacterial community diversity plays an important role in maintaining agricultural ecosystem stability, and improving crop resistance, crop growth and yield formation [42]. Plant diversity ensures soil bacterial community diversity and reduces the number of pathogenic microorganisms [43]. Qiao et al. [44] found that the intercropping of oats and vetch could improve the diversity of soil bacterial communities in the system. Conversely, consecutive monoculture of plants (cucumber and *Pseudostellaria heterophylla*) could decrease the diversity of the rhizosphere bacterial community [45,46]. Moreover, increased soil microbial diversity not only suppresses soil-borne diseases, but also improves soil nitrogen nutrient supply capacity [14]. Pankaj et al. [47] also found that significantly higher levels of nitrogen metabolic cycles could be observed in healthy soil as compared to soil with soil-borne diseases. It has been proven that legumes and non-legumes can efficiently utilize N sources in intercropping systems [48]. In this study, higher soil bacterial diversity indices were observed in the rhizosphere soil of both maize and peanut under belowground interaction intercropping (NS and SS). Moreover, genes related to soil N-cycling were improved by belowground interaction intercropping (NS and SS). These results demonstrated that the belowground interaction in intercropping systems improved soil bacterial community diversity and N-cycling bacteria, which were conducive to soil N nutrient supply capacity and soil health.

Based upon the discussion above, NS and SS show the same trends when compared with CS. Under NS treatment (no separation), the interactions between each crop include soil nutrition, microorganisms, root exudates exchanges, etc. The nylon net in SS treatment allowed root exudates, soil microorganisms, and soluble matter to flow across the net. Previous studies have demonstrated that plants release enormous amounts of chemicals through their roots, which affect soil microorganisms [49]. Because of the differences in the composition of root exudates, different crops and even genotypes can determine their unique soil microbial communities [15,50,51,52]. As such, we supposed that shifts in the soil microbial community in NS and SS were mainly due to root exudate exchanges in the intercropping.

## 4. Materials and Methods 

### 4.1. Experimental Design

The experiment was carried out in the experimental field and greenhouse of the College of Crop Sciences, Fujian Agricultural and Forestry University, Fuzhou, China (26°08′ N, 119°23′ E) in 2011 and 2012. In addition to monoculture (MS), maize (*Zea mays* L.) and peanut (*Arachis hypogaea* L.) were grown under three different intercropping treatments with the same canopy structure. Maize and peanut were planted with no separation intercropping (NS), semi-separation intercropping (SS) with a nylon net (50 µm), and complete separation intercropping (CS) with a plastic sheet (Figure S4). The experimental soil was sandy loam soil (pH value 5.5) containing total nitrogen 1.12 g·kg^−1^, total phosphorus 0.47 g·kg^−1^, total K 6.59 g·kg^−1^, available nitrogen 36.15 mg·kg^−1^, available phosphorus 8.23 mg·kg^−1^, and available K 30.46 mg·kg^−1^.

The yields of two crops were measured in 2011 and 2012 (Figure S4) in field experiments. The area for each pattern type (NS, SS, CS, and MS) was 16 m^2^ (4 × 4 m), with three replicates. The same field management and fertilization processes (including 180 kg·hm^−2^ CO(NH_2_)_2_, 52 kg·hm^−2^ Ca(H_2_PO_4_)_2_ and 75 kg·hm^−2^ KCl) were implemented in treatments during the whole experimental period. The pot experiment was carried out in 2012. Three peanut and one maize were grown in each pot with 13 kg of soil under three different intercropping treatments (NS, SS and CS), each with six replicates (Figure S5). Basal fertilizers were mixed in soil before planting, including (mg·kg^−1^ soil): N 100 (Ca (NO_3_)_2_·4H_2_O), P 150 (KH_2_PO_4_), K 150 (KCl), Mg 50 (MgSO_4_·7H_2_O), Cu 5(CuSO_4_·5H_2_O), and Zn 5(ZnSO_4_·7H_2_O).

### 4.2. Field Yields, Experimental Plant Property Determination and Soil Sampling

At maturity, five plants in each row were randomly selected in all treatments, and then the grain yields in each treatment were detected. The land equivalent ratio (LER) is the total land area of sole crops required to achieve the same yields as the intercrops.

LER = Y_im_/Y_m_ + Y_ip_/Y_p_ (1)

where Y_im_ and Y_m_ are the yields of intercropped and sole maize crops, and Y_ip_ and Y_p_ are the yields of intercropped and sole peanut crops, respectively. Intercropping systems exhibit yield advantages when LER > 1, while LER < 1 indicates yield disadvantages [53].

At the maize flowering stage (60 days after sowing) in the pot experiments, four maize and four peanut plants were randomly selected. The net photosynthetic rate of maize and peanut function leaves (the first fully expanded leaf from the top of the maize plant and last two or three leaves on the peanut main stem) were measured with Li-6400 (LI-COR, Lincoln, NE, USA) from 9:00 a.m. to 11:00 a.m. Peanut and maize were cut near the soil surface. The roots were carefully uprooted from the soil and gently shaken to remove loosely attached soil. The rhizosphere soils, at depths of 5~15 cm and closely attached to the roots, were carefully brushed down and collected, and then the rhizospheric soils were sieved by 0.9-mm mesh. Soil samples were separated into two parts: one part stored at −80 °C for soil DNA extraction, and other part stored at 4 °C for soil enzyme and soil nutrient analysis. The plant roots and peanut nodules were collected and immediately flushed with water. The flesh peanut nodules were detached from the roots and counted. These plants (shoots and roots) and nodules were first dried (105 °C for 30 min, 60 °C for 48 h), and then the biomasses were measured.

### 4.3. Measurement of Soil Nutrient and Enzymatic Activities

The measurement methods of soil nutrients (available nitrogen, phosphorus, and potassium) were proposed by Jackson [54]. Available nitrogen was measured by the alkaline hydrolysable method. Available phosphorus was measured by molybdenum blue method. Available potassium was extracted by incubating 5 g soil with 50 mL of 1 mol·L^−1^ CH_3_COONH_4_ (pH 7) for 30 min. The supernatant was filtered through the filter paper and then measured by flame photometry. The measurement methods of soil enzymes (urease, invertase, and acid PME) activities were proposed by Guan [29]. Urease (EC 3.5.1.5) activity was determined by incubating 5 g soil with 10 mL of 10% urea solution and 20 mL of citrate buffer (dissolved 184 g citric acid and 147.5 g KOH in 300 mL ddH_2_O respectively, combined the two solutions and then adjusted the pH to 6.7 with 1 mol·L^−1^ NaOH, and then diluted with ddH_2_O to 1 L) at 37 °C for 24 h. The formation of ammonium was measured by spectrophotometer at 578 nm and expressed as μg·g^−1^ soil·h^−1^. Soil invertase (EC 3.2.1.26) activity was determined by mixing 5 g soil with 15 mL of 8% sucrose and 5 mL of 66.7 mmol·L^−1^ phosphate buffer (pH 5.5) at 37 °C for 24 h. The supernatant was rapidly filtered through the filter paper and measured by 3,5-dinitrosalicylic acid colorimetric method (DNS method). Invertase activity expressed as μg glucose·g^−1^ soil·h^−1^. Acid PME (EC: 3.1.3.2) activity was determined by incubating 1 g soil with 4 mL of acidic buffer (20 mmol·L^−1^ Tris(hydroxymethyl)methyl aminomethane, 20 mmol·L^−1^ butenedioic acid, 14.6 mmol·L^−1^ citric acid, 20 mmol·L^−1^ boronic acid, pH 6.0), 0.2 mL of methylbenzene, and 1 mL of 0.05 mol·L^−1^ *para*-nitrophenyl phosphate (*p*NPP) at 37 °C for 24 h. The formation of *para*-nitrophenol was measured by spectrophotometer at 410 nm and expressed as μg·g^−1^ soil·h^−1^.

### 4.4. DNA Extraction and Terminal Restriction Fragment Length Polymorphism (T-RFLP) Analysis

The Soil Genomic DNA Extraction Kit (Bioer Technology Co., Ltd., Hangzhou, China) was used to extract soil whole genome DNA. DNA concentration was measured by Nanodrop and stored in −80 °C for T-RFLP [55] and quantification PCR analysis. Primers 27F-FAM (5′-AGAGTTTGATCCTGGCTCAG-3′) and 1492R (5′-GGTTACCTTGTTACGACTT-3′) with 6-carboxyfurescein-label were used to amplify the bacterial 16S rRNA gene. The PCR reaction mixture (50 μL in final volume) included 25 μL Taq PCR Mix (2×) (TianGen Biotech Co., Ltd., Beijing, China), 1 μL of each primer (10 pmol·mL^−1^), 1 μL 0.1% BSA (Bovine Serum Albumin), 20 ng DNA template, and ddH_2_O. The PCR program was as follows: 5 min at 95 °C, followed by 35 cycles of 95 °C for 1 min, 60 °C for 90 s, and 72 °C for 90 s, and then 1 cycle of 72 °C for 10 min. To purify PCR products, 1.2% agarose gel electrophoresis and the Gel Extraction Kit (OMEGA Bio-Tek, Norcross, GA, USA) were used. The purified PCR products were digested separately with four enzymes (*Msp*I, *Hae*III, *Afa*I and *Alu*I) [55]. The digestion mixture (15 μL in final volume) included 4 µL of enzyme buffer, 1.5 µL of restriction endonuclease, 8 µL of PCR products and ddH_2_O. The endonuclease digestion products were determined by the ABI 3730xl DNA sequencer (Applied Biosystems, Foster City, CA, USA). PCR amplification and enzyme digestion took place in dark conditions to avoid fluorescence decay. Gene Marker software Version 1.2 (SoftGenetics LLC, State College, PA, USA) was used to analyze T-RFLP profiles. Lengths of terminal restriction fragments (T-RFs) between 30 and 600 bp were selected for further analysis. The T-RFs measuring ±1 bp were combined and considered as the same operational taxonomic units [56]. Based on the T-RFs, bacteria were identified by the phylogenetic assignment tool (PAT) [57]. Only three or four restriction enzyme T-RF lengths matching phylogenetic assignments were used in this study.

### 4.5. Quantification PCR of Bacterial Communities

Quantification PCR (qPCR) was used to analyze the bacterial genes (*nifH*, *amoA* (AOA), *amoA* (AOB), *narG*, *nirK*, *nirS,* and *nosZ*), which encode the key enzymes in nitrogen cycling (Table S2). *AmoA* (AOB) and *amoA* (AOA) encode ammonia monooxygenase; *narG* encodes membrane-bound nitrate reductase; *nirK* and *nirS* encode nitrite reductase; *nosZ* encodes nitrous oxide reductase; and *nifH* encode the nitrogenase. Meanwhile, the qPCR of the main bacterial phylum (α-Proteobacteria, β-Proteobacteria, Firmicutes and Actinobacteria) and beneficial bacteria (*Bacillus*, *Burkholderia*, *Pseudomonas*, *Rhizobium*) measured by the methods are described in Table S2. qPCR reaction mixture (15 μL in final volume) included 0.5 μL of each primer (10 μM), 7.5 μL (2×) SYBR green I SuperReal Premix (TIANGEN, Beijing, China), and template DNA (20 ng of total soil DNA or plasmid DNA for standard curves).

### 4.6. Statistical Analyses

SPSS V11.5 software (IBM Corporation, Chicago, IL, USA) was used for statistical analysis, and ANOVA (analysis of variance) was used to determine the significance of difference with the LSD’s test (*p* < 0.05). PRIMER V5 software (PRIMER-E Ltd., Plymouth, UK) was used for non-metric multidimensional scaling (NMDS) and similarity percentage analysis (SIMPER) of T-RFLP data. NMDS is a superior analysis method for investigating microbial community data [58], and is considered an accurate and reliable method of analysis when Kruskal’s stress value <0.1. The similarities of microbial communities were evaluated by NMDS in this study. The contribution (%) of each T-RF to the dissimilarity between samples was carried out by means of similarity percentage analysis [58].

## 5. Conclusions

The advantages of maize/peanut intercropping can be attributed to belowground interactions. Intercropping of maize and peanut improved the soil nutrient supply capacity and soil health. This improvement was driven by the shifts of the soil microbial community, including the improvement in the abundance of beneficial bacteria (i.e., *Bacillus*, *Burkholderia*, *Pseudomonas*, and *Rhizobium* etc.), bacterial diversity, and N-cycling bacteria. Besides plant nutrition, our study suggested that the soil microbial community could be a key factor for reasonable intercropping, and that the interaction of root exudates may play a key role in modifying the soil microbial community. This study also provided a clue to solving problems stemming from excessive monoculture in current agricultural production. Additional work is needed to explore the interactions of root exudates in intercropping, and the mechanisms of how root exudates shape the soil microbial community.

## Figures and Tables

**Table 1:** Maize and peanut yields under different intercropping treatments.

Treatments	Maize Yield (Mg·ha^−1^)	Peanut Yield (Mg·ha^−1^)	LER
2011			
NS	8.62 ± 0.39a	3.42 ± 0.14a	1.31
SS	9.00 ± 0.34a	3.32 ± 0.14ab	1.33
CS	4.69 ± 0.29b	3.14 ± 0.06b	0.95
MS	4.70 ± 0.30b	3.38 ± 0.07ab	1
2012			
NS	10.22 ± 0.33a	3.69 ± 0.35a	1.11
SS	9.95 ± 0.22a	3.61 ± 0.15a	1.08
CS	8.15 ± 0.30b	3.40 ± 0.17a	0.97
MS	8.06 ± 0.39b	3.61 ± 0.27a	1

NS: non-separation intercropping; SS: semi-separation intercropping; CS: complete separation intercropping; MS: monoculture treatment; LER: land equivalent ratios. Different letters show significant differences determined by the LSD (least significant difference) test (*p <* 0.05, *n* = 3).

**Table 2:** Plant biomass, root nodulation, and net photosynthetic rate of both crops in three different intercropping treatments.

Treatments	Biomass (g)	Shoot Biomass (g)	Root Biomass (g)	Pn (μmol CO_2_·m^−2^·s^−1^)	Nodule Number per Plant	Dry Weight per Nodule (mg)
Maize						
NS	118.40 ± 6.26a	95.13 ± 4.71a	23.27 ± 1.55a	40.38 ± 1.81a	/	/
SS	120.22 ± 5.56a	94.95 ± 4.81a	25.27 ± 0.75a	40.23 ± 3.52a	/	/
CS	94.19 ± 2.98b	76.16 ± 2.58b	18.03 ± 0.40b	34.48 ± 1.08b	/	/
Peanut						
NS	13.95 ± 0.99a	12.36 ± 0.76a	1.59 ± 0.23a	26.60 ± 1.53a	528.33 ± 25.42a	0.51 ± 0.03ab
SS	14.60 ± 1.07a	12.83 ± 0.70a	1.77 ± 0.37a	27.60 ± 1.63a	626.00 ± 40.00a	0.57 ± 0.06a
CS	14.30 ± 0.72a	13.17 ± 0.66a	1.13 ± 0.06b	24.98 ± 1.67a	310.00 ± 50.20b	0.43 ± 0.04b

NS: non-separation intercropping; SS: semi-separation intercropping; CS: complete separation intercropping; Pn: net photosynthetic rate. Different letters show significant differences determined by the LSD test (*p <* 0.05, *n* = 3).

**Table 3:** Diversity analysis of rhizosphere soil bacterial communities under different intercropping patterns.

Treatments	Simpson Index (J)	Shannon-Wiener Index (H)
Maize		
NS	0.988a	5.921a
SS	0.984b	5.84b
CS	0.982c	5.71c
Peanut		
NS	0.985a	5.826a
SS	0.986a	5.862a
CS	0.982b	5.596c

NS: non-separation intercropping; SS: semi-separation intercropping; CS: complete separation intercropping. Different letters indicate significant differences (LSD test, *p* < 0.05, *n* = 3).

**Table 4:** Top terminal restriction fragments (T-RFs) with 20% cumulative contribution to the dissimilarity between belowground interaction (NS and SS) and complete separation (CS).

Contribution (%)	TRFLP-PAT Assignment	Relative Abundance (%)			Functions
		NS	SS	CS	
Maize					
4.63	*Brevibacillus brevis* (D78457)	3.53	3.63	0	Improving root growth, nodulation and pathogen antagonism
3.33	*Paenibacillus* sp.	2.8	2.35	0	N-fixation and pathogen antagonism
1.87	*Bacillus* sp.	6	5.65	4.38	Improving nodulation, P-solubilization and pathogen antagonism
1.68	Clone OCS155 (AF001652)	1.25	0	3.46	Unknown
1.58	No Match	0.85	0.96	0.18	Unknown
1.28	*Polyangium* sp.	0.76	0.79	1.62	C cycle
1.21	*Bacillus subtilis* (AL009126)	4.99	4.77	3.95	Improving nodulation, P-solubilization and pathogen antagonism
1.19	Clone T33 (Z93960)	1.33	0.32	1.55	Unknown
1.12	*Pseudomonas* sp.	3.64	4.32	3.26	P-solubilizing and pathogen antagonism
1.08	*Acidosphaera* (D86512)	1.4	0.8	1.02	Unknown
1.02	*Sphingomonas* sp.	1.52	1.24	1.89	C cycle
Peanut					
2.23	*Bacillus* sp.	8.33	8.89	5.81	Improving nodulation, P-solubilization and pathogen antagonism
1.98	*Burkholderia* sp.	5.78	5.62	3.49	Legume N-fixing symbiont. Pathogen antagonism and plant growth promotion
1.81	*Clostridium* sp.	1.53	1.32	3.98	C cycle
1.6	*Pseudomonas* sp.	1.25	1.44	0.62	P-solubilization and pathogen antagonism
1.59	str. AS2988.(AF060671)	1.4	0.94	0	Unknown
1.5	*Nocardia crassostrae* (U92800)	0.99	1.13	3.44	Unknown
1.31	clone Sva0556.	0.49	0	1.8	Unknown
1.29	*Brevibacillus brevis* (D78457)	3.45	3.08	2.65	Improving root growth, nodulation and pathogen antagonism
1.2	No match	0	0	0.87	Unknown
1.02	*Xylophilus ampelinus* (AF078758)	0.77	1.38	2.98	Plant pathology
0.99	*Cytophaga lytica* (M62796)	1.24	1.24	0	C cycle
0.95	*Mesorhizobium loti* (D14514)	2.71	3.3	1.81	Legume nodulation and N fixation
0.9	*Rhizobium hainanense* (U71078)	1.53	1.7	0.89	Legume nodulation and N fixation
0.86	*Afipia clevelandensis* (M69186)	1.9	2.3	2.5	Nitrification
0.86	*Sphingomonas* sp. (U52146)	1.35	1.59	0.66	C cycle

NS: non-separation intercropping; SS: semi-separation intercropping; CS: complete separation intercropping; TRFLP-PAT: terminal restriction fragment length polymorphism-phylogenetic assignment tool. The references of bacterial functions are shown in Table S1.

## Supplementary Materials

Supplementary materials can be found at http://www.mdpi.com/1422-0067/19/2/622/s1.

## Abbreviations

NS	Non-separation intercropping
SS	Semi-separation intercropping
CS	Complete separation intercropping
MS	Monoculture
NM	Non-separation intercropping maize
SM	Semi-separation intercropping maize
CM	Complete separation intercropping maize
NP	Non-separation intercropping peanut
SP	Semi-separation intercropping peanut
CP	Complete separation intercropping peanut
LER	Land equivalent ratio
