# Supplementary material for: Belowground Interactions Impact the Soil Bacterial Community, Soil Fertility, and Crop Yield in Maize/Peanut Intercropping Systems
Source: Int J Mol Sci. 2018 Feb 22;19(2):622. doi: 10.3390/ijms19020622 (PMC5855844; doi:10.3390/ijms19020622)
Supplement: Supplementary file 1 [file ijms-19-00622-s001.pdf]

Supplementary Materials: Belowground Interactions Impact the Soil Bacterial Community, Soil Fertility and Crop Yield in Maize/Peanut Intercropping Systems

Qisong Li, Jun Chen, Linkun Wu, Xiaomian Luo, Na Li, Yasir Arafat, Sheng Lin, Wenxiong Lin

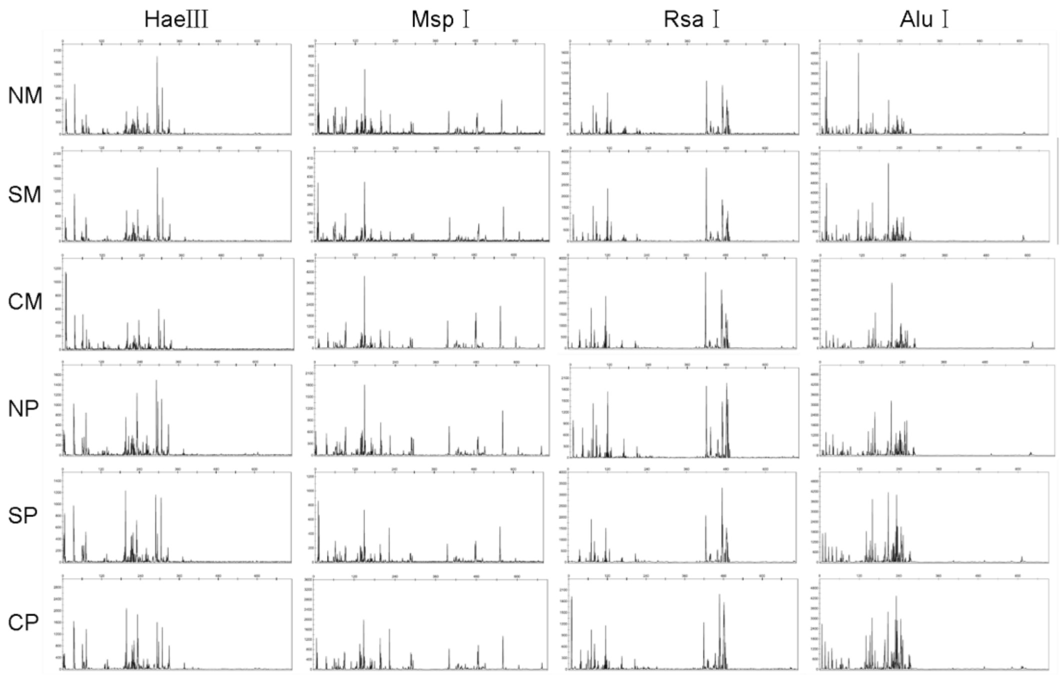

Figure S1. T-RFLP profiles of bacterial 16s rRNA genes amplified from different treatments soils for each restriction enzyme. NM: non-separated maize; SM semi-separated maize; CM: completely separated maize; NP: non-separated peanut SP: semi-separated peanut; CP: completely separated peanut.

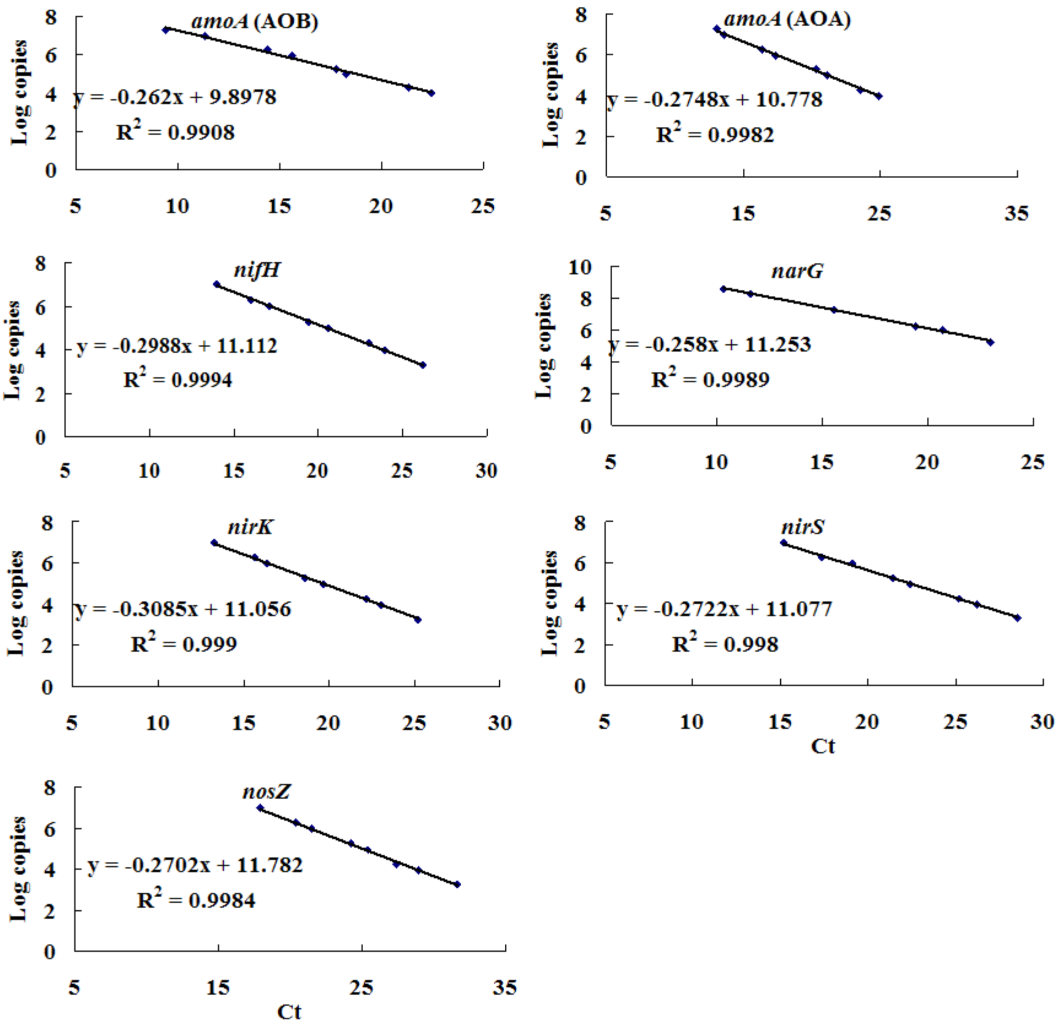

Figure S2. Standard curves for the quantification of genes involving in soil N cycling.

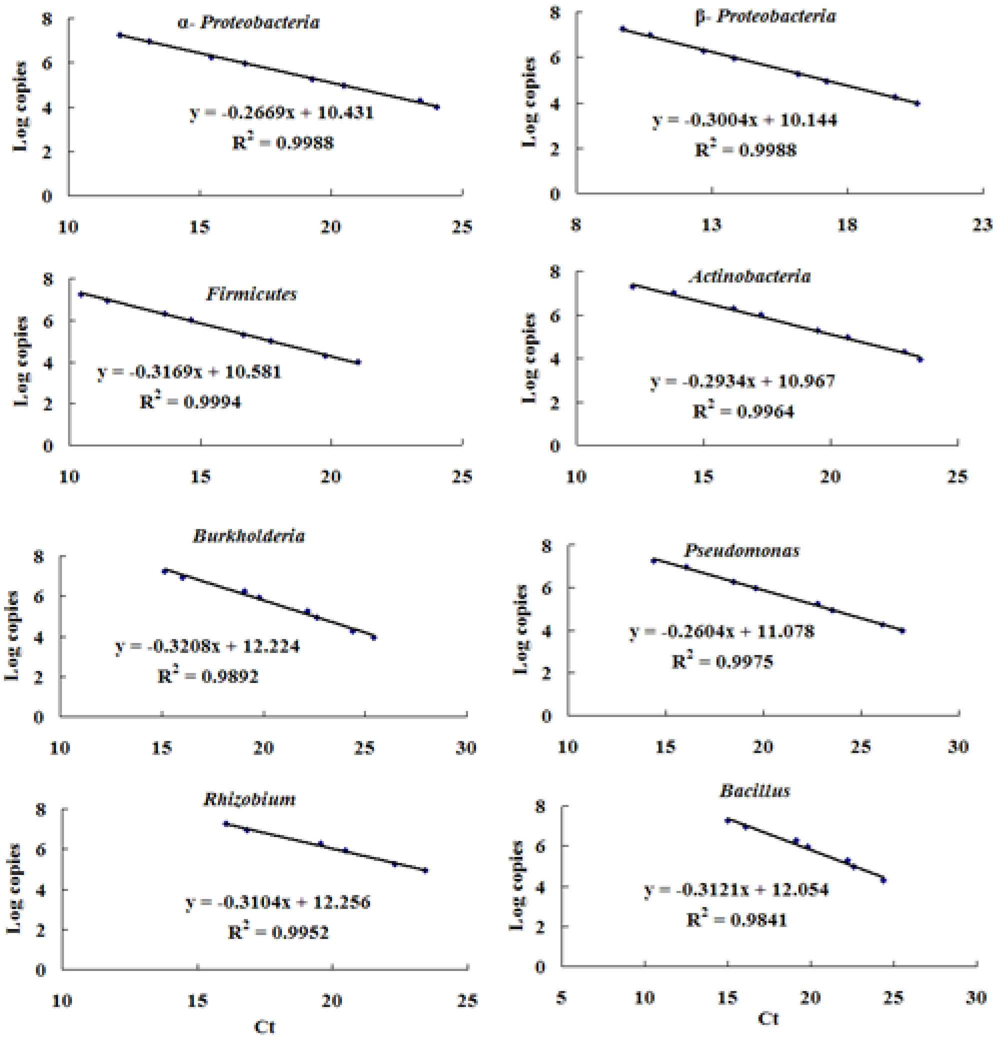

Figure S3. Standard curves for the quantification of genes which involved in major microbial communities under different intercropping patterns.

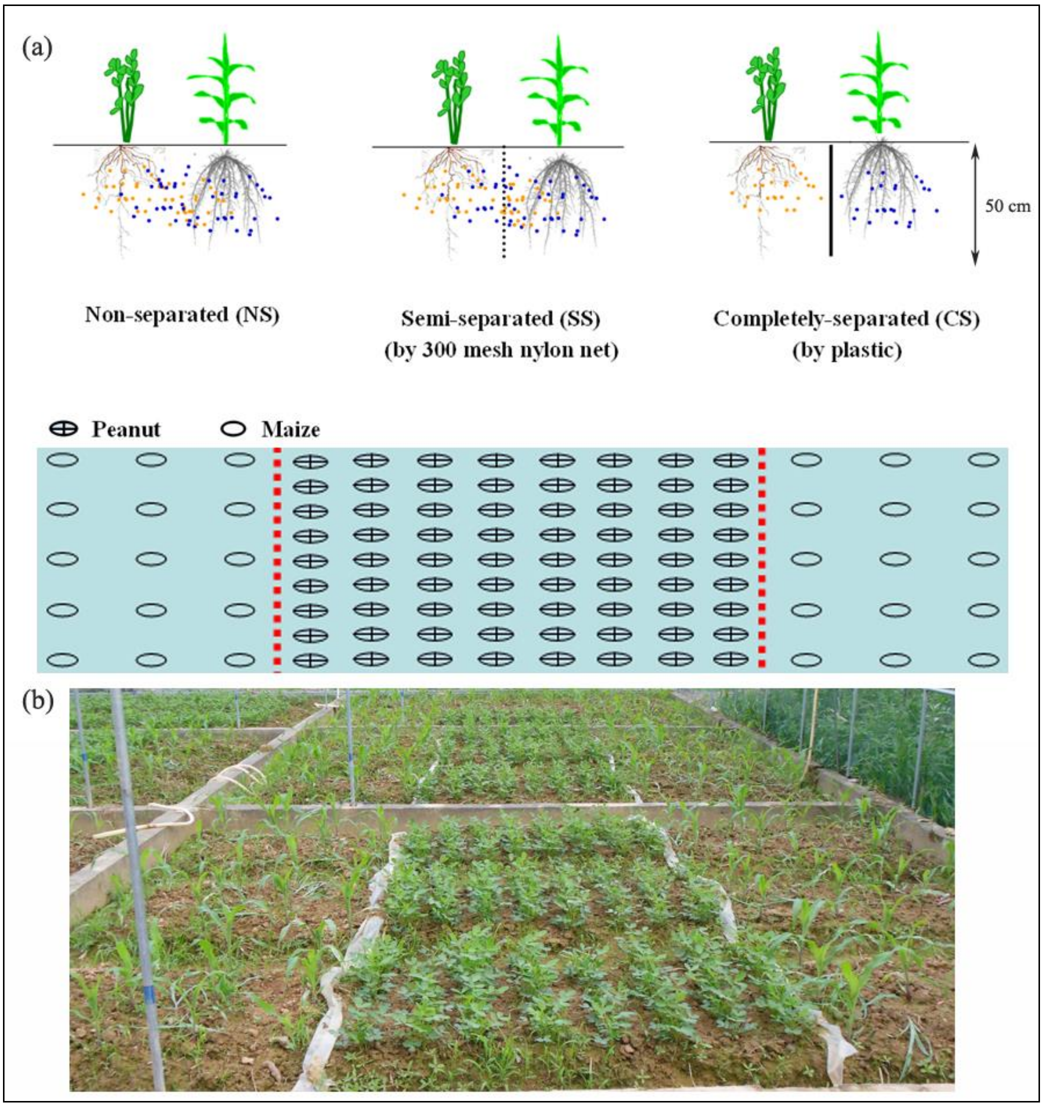

Figure S4. Field experimental designs regard maize and peanut intercropping systems. Field experimental designs (**a**) and actual growth status (**b**) under maize and peanut intercropping systems. NS: non-separation treatment; SS: semi-separation treatment; CS: completely separated treatment. Row space was 20 and 35 cm for peanut and maize treatment, respectively. Interplant space was 20 and 35 cm for peanut and maize treatment, respectively. The distance between wheat strip and maize strip was 22.5 cm. The row ratio of maize and peanut in all intercroppings were 3:8. Root barrier depth was 50 cm.

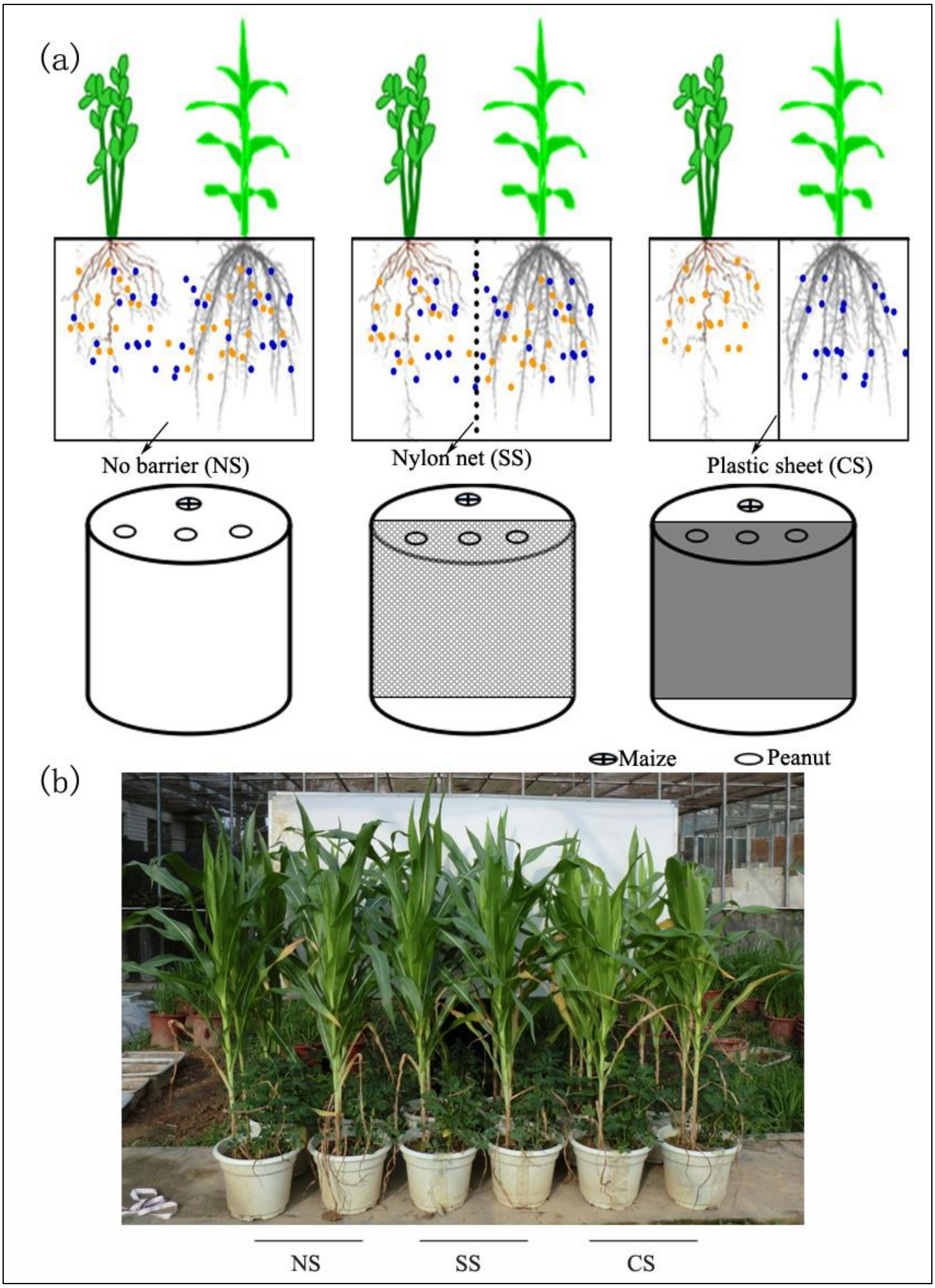

Figure S5. Pot experimental designs and actual growth status regard maize and peanut intercropping systems. Pot experimental designs (**a**) and actual growth status (**b**) under maize and peanut intercropping systems. NS: non-separation treatment; SS: semi-separation treatment; CS: complete separation treatment. Black and blue spots indicate root exudates and soil microorganisms of peanut and maize, respectively.

Table S1. Top terminal restriction fragments (T-RFs) with 20% cumulative contribution to the dissimilarity between belowground interaction intercropping (NS and SS) and completely separated intercropping (CS).

**Enzymes**				**TRFLP-PAT assignment**	**Reference**
***Msp* I**	***Hae* III**	***Afa* I**	***Alu* I**		
				Maize	
122	219	444	234	*Brevibacillus brevis* (D78457)	[1]
150	235	894	75	*Paenibacillus* sp.	[2]
153	231	456	73	*Bacillus* sp.	[2]
171	330	459	64	clone OCS155 (AF001652)	
	123			No Match	
78	75	488	217	*Polyangium* sp.	[3]
145	309	456	73	*Bacillus subtilis* (AL009126)	[2,4]
488	217	427	148	clone T33 (Z93960)	
492	39	72	236	*Pseudomonas* sp.	[3,5]
64	63	425	144	*Acidosphaera* (D86512)	
150	227	422	250	*Sphingomonas* sp.	[6]
				Peanut	
153	231	456	73	*Bacillus* sp.	[2]
487	197	562	152	*Burkholderia* sp.	[7,8]
520	222	451	237	*Clostridium* sp.	[9]
492	39	72	236	*Pseudomonas* sp.	[3,5]
139	206	451	232	str. AS2988.(AF060671)	
159	67	78	232	*Nocardia crassostrae* (U92800)	
509	218	58	67	clone Sva0556.	
294	219	444	234	*Brevibacillus brevis* (D78457)	[1]
	65			No Match	
488	217	427	148	*Xylophilus ampelinus* (AF078758)	[10]
91	282	310	73	*Cytophaga lytica* (M62796)	[11]
125	221	678	138	*Mesorhizobium loti* (D14514)	[12]
401	191	824	206	*Rhizobium hainanense* (U71078)	[13]
152	194	110	209	*Afipia clevelandensis* (M69186)	[14]
150	293	422	208	*Sphingomonas* sp. (U52146)	[6]

Table S2. PCR primers and thermal cycling conditions used for quantification of different genes.

Primers	Sequence (5′ – 3′)	Thermal conditions
NifH [15]<br>nifH-F<br>nifH-R	CCT ACG GGA GGC AGC AG<br>ATT ACC GCG GCT GCT GGC A	95°C, 15 min, 1 cycle<br>95°C for 15 s, 60°C for 30 s, 72°C for 30 s, 80°C for 15 s, 40 cycles<br>95°C for 15 s, 60 to 95°C, 1 cycle
NarG [16]<br>narG1960m2F<br>narG2050m2R	TAY GTS GGG CAG GAR AAA CTG<br>CGT AGA AGA AGC TGG TGC TGT T	95°C, 15 min, 1 cycle<br>95°C for 15 s, 65 to 60°C for 30 s (-1°C by cycle), 72°C for 30 s, 80°C for 15 s, 6 cycles<br>95°C for 15 s, 60°C for 30 s, 72°C for 30 s, 80°C for 15 s, 40 cycles<br>95°C for 15 s, 60 to 95°C, 1 cycle

NirK [17]<br>nirK876<br>nirK1040	ATY GGC GGV CAY GGC GA<br>GCC TCG ATC AGR TTR TGG TT	95°C, 15 min, 1 cycle<br>95°C for 15 s, 63 to 58°C for 30 s (-1°C by cycle), 72°C for 30 s, 80°C for 15 s, 6 cycles<br>95°C for 15 s, 60°C for 30 s, 72°C for 30 s, 80°C for 15 s, 40 cycles<br>95°C for 15 s, 60 to 95°C, 1 cycle
nirS [18]<br>nirSCd3aFm<br>nirSR3cdm	AAC GYS AAG GAR ACS GG<br>GAS TTC GGR TGS GTC TTS AYG AA	95°C, 15 min, 1 cycle<br>95°C for 15 s, 65 to 60°C for 30 s (-1°C by cycle), 72°C for 30 s, 80°C for 15 s, 6 cycles<br>95°C for 15 s, 60°C for 30 s, 72°C for 30 s, 80°C for 15 s, 40 cycles<br>95°C for 15 s, 60 to 95°C, 1 cycle
NosZ [18]<br>nosZ2F<br>nosZ2R′	CGC RAC GGC AAS AAG GTS MSS GT<br>CAK RTG CAK SGC RTG GCA GAA	95°C, 15 min, 1 cycle<br>95°C for 15 s, 65 to 60°C for 30 s (-1°C by cycle), 72°C for 30 s, 80°C for 15 s, 6 cycles<br>95°C for 15 s, 60°C for 30 s, 72°C for 30 s, 80°C for 15 s, 40 cycles<br>95°C for 15 s, 60 to 95°C, 1 cycle
amoA(AOB) [19]<br>amoA-1F<br>amoA-2R	GGG GTT TCT ACT GGT GGT<br>CCC CTC KGS AAA GCC TTC TTC	95°C, 10 min, 1 cycle<br>94°C for 45 s, 58°C for 45 s, 72°C for 45 s, 39 cycles<br>95°C for 15 s, 60°C for 30 s, to 95°C for 15 s, 1 cycle
amoA(AOA) [20]<br>19F<br>CrenamoA616r48x	ATG GTC TGG CTW AGA CG<br>GCC ATC CAB CKR TAN GTC CA	95°C, 10 min, 1 cycle<br>94°C for 45 s, 55°C for 45 s, 72°C for 45 s, 39 cycles<br>95°C for 15 s, 60°C for 30 s, to 95°C for 15 s, 1 cycle
Actinobacteria [21]<br>Actino235<br>Eub518	CGC GGC CTA TCA GCT TGT TG<br>ATT ACC GCG GCT GCT GG	95°C, 15 min, 1 cycle<br>95°C for 1 min, 60°C for 30 s, 72°C for 1 min, 40 cycles
Alphaproteobacteria [21]<br>Eub338<br>Alfa685	ACT CCT ACG GGA GGC AGC AG<br>TCT ACG RAT TTC ACC YC TAC	95°C, 15 min, 1 cycle<br>95°C for 1 min, 60°C for 30 s, 72°C for 1 min, 40 cycles
Betaproteobacteria [21]<br>Eub338<br>Bet680	ACT CCT ACG GGA GGC AGC AG<br>TCA CTG CTA CAC GYG	95°C, 15 min, 1 cycle<br>95°C for 1 min, 60°C for 30 s, 72°C for 1 min, 40 cycles
Firmicutes [21]<br>Lgc353<br>Eub518	GCA GTA GGG AAT CTT CCG<br>ATT ACC GCG GCT	95°C, 15 min, 1 cycle<br>95°C for 1 min, 60°C for 30 s, 72°C for 1 min, 40 cycles

	GCT GG	
Pseudomonas sp. [21]<br>PsF<br>PsR	TTA GCT CCA CCT CGC GGC<br>GGT CTG AGA GGA TGA TCA GT	95°C, 15 min, 1 cycle<br>95°C for 1 min, 64°C for 30 s, 72°C for 1 min, 40 cycles
Burkholderia sp. [21]<br>Burk3<br>BurkR	CTG CGA AAG CCG GAT<br>TGC CAT ACT CTA GCY YGC	95°C, 15 min, 1 cycle<br>95°C for 1 min, 64°C for 30 s, 72°C for 1 min, 40 cycles
Bacillus sp. [21]<br>BacF<br>1378	GGG AAA CCG GGG CTA ATA CCG GAT<br>CGG TGT GTA CAA GGC CCG GGA ACG	95°C, 15 min, 1 cycle<br>95°C for 1 min, 63°C for 30 s, 72°C for 1 min, 40 cycles
Rhizobium sp. [22]<br>F979<br>R1264	CCC GGC TAC YTG CAG AGA TG<br>TAG CTC ACA CTC GCG TGC TC	94°C, 15 min, 1 cycle<br>94°C for 20 s, 60 °C for 30 s, 72°C for 30 s, 40 cycle

References

1. Vivas, A.; Barea, J.; Azcón, R. Interactive effect of brevibacillus brevis and glomus mosseae, both isolated from cd contaminated soil, on plant growth, physiological mycorrhizal fungal characteristics and soil enzymatic activities in cd polluted soil. *Environ. Pollut.* **2005**, *134*, 257–266.
2. McSpadden Gardener, B.B. Ecology of bacillus and paenibacillus spp. In agricultural systems. *Phytopathology* **2004**, *94*, 1252–1258.
3. Osman, K.T. Biological properties of soils. **2013**, *8*, 49–65.
4. Figueiredo, M.; Martinez, C.; Burity, H.; Chanway, C. Plant growth-promoting rhizobacteria for improving nodulation and nitrogen fixation in the common bean (phaseolus vulgaris l.). *World J. Microbiol. Biotechnol.* **2008**, *24*, 1187–1193.
5. Nautiyal, C.S. An efficient microbiological growth medium for screening phosphate solubilizing microorganisms. *FEMS Microbiol. Lett.* **1999**, *170*, 265–270.
6. Leys, N.M.; Ryngaert, A.; Bastiaens, L.; Verstraete, W.; Top, E.M.; Springael, D. Occurrence and phylogenetic diversity of sphingomonas strains in soils contaminated with polycyclic aromatic hydrocarbons. *Appl. Environ. Microbiol.* **2004**, *70*, 1944–1955.
7. Bontemps, C.; Elliott, G.N.; Simon, M.F.; Dos Reis Junior, F.B.; Gross, E.; Lawton, R.C.; Neto, N.E.; De FÁTIMA LOUREIRO, M.; De Faria, S.M.; Sprent, J.I. Burkholderia species are ancient symbionts of legumes. *Mol. Ecol.* **2010**, *19*, 44–52.
8. Brahmaprakash, G.; Sahu, P.K.; Lavanya, G.; Nair, S.S.; Gangaraddi, V.K.; Gupta, A. Microbial functions of the rhizosphere. In *Plant-microbe interactions in agro-ecological perspectives*, Springer: 2017; pp 177-210.
9. Zhu, Y.; Wu, Z.; Yang, S.-T. Butyric acid production from acid hydrolysate of corn fibre by clostridium tyrobutyricum in a fibrous-bed bioreactor. *Process Biochem.* **2002**, *38*, 657–666.
10. Dreo, T.; Gruden, K.; Manceau, C.; Janse, J.; Ravnikar, M. Development of a real-time pcr-based method for detection of xylophilus ampelinus. *Plant Pathol.* **2007**, *56*, 9–16.
11. Singh, S.; Nain, L. In *Microorganisms in the conversion of agricultural wastes to compost*, Proc Indian Natn Sci Acad, 2014; pp 473-481.
12. Laranjo, M.; Alexandre, A.; Oliveira, S. Legume growth-promoting rhizobia: An overview on the mesorhizobium genus. *Microbiol. Res.* **2014**, *169*, 2–17.

13. Chen, W.-X.; Tan, Z.-Y.; Gao, J.-L.; Li, Y.; Wang, E.-T. Rhizobium hainanense sp. Nov., isolated from tropical legumes. *Int. J. Syst. Evol. Microbiol.* **1997**, *47*, 870–873.
14. Bock, E.; Wagner, M. Oxidation of inorganic nitrogen compounds as an energy source. In *The prokaryotes*, Springer: 2006; pp 457-495.
15. Rösch, C.; Mergel, A.; Bothe, H. Biodiversity of denitrifying and dinitrogen-fixing bacteria in an acid forest soil. *Appl. Environ. Microbiol.* **2002**, *68*, 3818–3829.
16. López-Gutiérrez, J.C.; Henry, S.; Hallet, S.; Martin-Laurent, F.; Catroux, G.; Philippot, L. Quantification of a novel group of nitrate-reducing bacteria in the environment by real-time pcr. *J. Microbiol. Methods* **2004**, *57*, 399–407.
17. Henry, S.; Baudoin, E.; López-Gutiérrez, J.C.; Martin-Laurent, F.; Brauman, A.; Philippot, L. Quantification of denitrifying bacteria in soils by nirk gene targeted real-time pcr. *J. Microbiol. Methods* **2004**, *59*, 327–335.
18. Throbäck, I.N.; Enwall, K.; Jarvis, Å.; Hallin, S. Reassessing pcr primers targeting nirs, nirk and nosz genes for community surveys of denitrifying bacteria with dgge. *FEMS Microbiol. Ecol.* **2004**, *49*, 401–417.
19. Rotthauwe, J.-H.; Witzel, K.-P.; Liesack, W. The ammonia monooxygenase structural gene amoa as a functional marker: Molecular fine-scale analysis of natural ammonia-oxidizing populations. *Appl. Environ. Microbiol.* **1997**, *63*, 4704–4712.
20. Leininger, S.; Urich, T.; Schloter, M.; Schwark, L.; Qi, J.; Nicol, G.; Prosser, J.; Schuster, S.; Schleper, C. Archaea predominate among ammonia-oxidizing prokaryotes in soils. *Nature* **2006**, *442*, 806–809.
21. Fierer, N.; Jackson, J.A.; Vilgalys, R.; Jackson, R.B. Assessment of soil microbial community structure by use of taxon-specific quantitative pcr assays. *Appl. Environ. Microbiol.* **2005**, *71*, 4117–4120.
22. Macdonald, C.A.; Clark, I.M.; Hirsch, P.R.; Zhao, F.-J.; McGrath, S.P. Development of a real-time pcr assay for detection and quantification of rhizobium leguminosarum bacteria and discrimination between different biovars in zinc-contaminated soil. *Appl. Environ. Microbiol.* **2011**, *77*, 4626–4633.

© 2018 by the authors. Submitted for possible open access publication under the terms and conditions of the Creative Commons Attribution (CC BY) license (http://creativecommons.org/licenses/by/4.0/).
